# Using Beta-Version mHealth Technology for Team-Based Care Management to Support Stroke Prevention: An Assessment of Utility and Challenges

**DOI:** 10.2196/resprot.7106

**Published:** 2017-05-23

**Authors:** Magaly Ramirez, Shinyi Wu, Gery Ryan, Amytis Towfighi, Barbara G Vickrey

**Affiliations:** ^1^ Fielding School of Public Health Department of Health Policy and Management University of California, Los Angeles Los Angeles, CA United States; ^2^ Suzanne Dworak-Peck School of Social Work University of Southern California Los Angeles, CA United States; ^3^ Edward R Roybal Institute on Aging University of Southern California Los Angeles, CA United States; ^4^ Daniel J Epstein Department of Industrial and Systems Engineering University of Southern California Los Angeles, CA United States; ^5^ RAND Corporation Santa Monica, CA United States; ^6^ Keck School of Medicine Department of Neurology University of Southern California Los Angeles, CA United States; ^7^ Rancho Los Amigos National Rehabilitation Center Downey, CA United States; ^8^ Department of Neurology Icahn School of Medicine Mount Sinai New York, NY United States

**Keywords:** community health workers, stroke, patient care management, patient care team, mobile devices, mobile applications, health care information systems

## Abstract

**Background:**

Beta versions of health information technology tools are needed in service delivery models with health care and community partnerships to confirm the key components and to assess the performance of the tools and their impact on users. We developed a care management technology (CMT) for use by community health workers (CHWs) and care managers (CMs) working collaboratively to improve risk factor control among recent stroke survivors. The CMT was expected to enhance the efficiency and effectiveness of the CHW-CM team.

**Objective:**

The primary objective was to describe the Secondary Stroke Prevention by Uniting Community and Chronic Care Model Teams Early to End Disparities (SUCCEED) CMT and investigate CM and CHW perceptions of the CMT’s usefulness and challenges for team-based care management.

**Methods:**

We conducted qualitative interviews with all users of the beta-version SUCCEED CMT, namely two CMs and three CHWs. They were asked to demonstrate and describe their perceptions of the CMT’s ease of use and usefulness for completing predefined key care management activities. They were also probed about their general perceptions of the CMT’s information quality, ease of use, usefulness, and impact on CM and CHW roles. Interview transcripts were coded using a priori codes. Coded excerpts were grouped into broader themes and then related in a conceptual model of how the CMT facilitated care management. We also conducted a survey with 14 patients to obtain their perspective on CHW tablet use during CHW-patient interactions.

**Results:**

Care managers and community health workers expressed that the CMT helped them keep track of patient interactions and plan their work. It guided CMs in developing and sharing care plans with CHWs. For CHWs, the CMT enabled electronic collection of clinical assessment data, provided decision support, and provided remote access to patients’ risk factor values. Long loading times and downtimes due to outages were the most significant challenges encountered. Additional issues included extensive use of free-text responses and manual data transfer from the electronic medical record. Despite these challenges, patients overall did not perceive the tablet as interfering with CHW-patient interactions.

**Conclusions:**

Our findings suggest useful functionalities of CMTs supporting health care and community partners in collaborative chronic care management. However, usability issues need to be addressed during the development process. The SUCCEED CMT is an initial step toward the development of effective health information technology tools to support collaborative, team-based models of care and will need to be modified as the evidence base grows. Future research should assess the CMT’s effects on team performance.

## Introduction

Health information technology (HIT) has the potential to facilitate the delivery of collaborative, team-based approaches to chronic illness care [1]. Although such chronic care models—which often involve health care and community partnerships—can lead to improvements in patient care and health outcomes [2], implementation requires streamlined information processing, communication, and management. Bauer et al [3] described HIT capabilities that may increase the effectiveness and efficiency of chronic care delivery. These include HIT tools that enable users to create care plans that can be shared among members of the care team, provide clinical decision support, incorporate treatment algorithms, monitor patients’ progress, alert providers of patients in need, and track patient visits and outreach efforts. Despite the promise of HIT to support team-based models of chronic disease care, a systematic review by Dorr et al [4] found that the most common intended users of HIT tools tend to be physicians. HIT tools for use by health care and community partners to support improved care coordination for chronic conditions have been understudied. Investigations of HIT in service delivery models with health care and community partnerships are needed to build the evidence base of how technology-enabled models of team care can improve team performance and reduce costs. To begin, beta versions of the tools need to be developed to confirm the key components and assess the performance of the tools and their impact on users.

A research team consisting of clinicians, implementation scientists, systems engineering and human factors specialists, and end users—care managers (CMs) and community health workers (CHWs)—worked with a software company to develop a care management technology (CMT) for an intervention that uses a team of care providers to collaboratively improve risk factor control among stroke survivors. This technology-facilitated intervention—called Secondary Stroke Prevention by Uniting Community and Chronic Care Model Teams Early to End Disparities (SUCCEED)—is being tested for its impact on secondary stroke prevention and cost-effectiveness analysis in a randomized controlled trial with a multiethnic, underresourced population in Los Angeles County, California [5]. The fundamental purpose of the CMT in the SUCCEED program was to facilitate more effective and efficient care management and care coordination among care team members and to facilitate encounters with patients. This paper presents results from our investigation of perceptions of care team members regarding the usefulness and challenges of the CMT for recurrent stroke prevention care management. We also discuss our experience designing, developing, and implementing the CMT and implications for researchers interested in conducting research studies using CMTs. We intended for the SUCCEED CMT to serve as an initial step toward the development of HIT tools to facilitate effective and efficient collaborative, team-based models of chronic illness care.

### SUCCEED Program Description

Recent stroke survivors enrolled in the SUCCEED program received care for 1 year from a care team consisting of a CM, who was either a nurse practitioner or physician assistant, and a CHW. The goal of the care team was to improve patients’ control of stroke risk factors (ie, blood pressure, cholesterol, diabetes, diet, physical activity, smoking, alcohol, and illicit drug use) and to assess for and address complications including social isolation and depression. The team provided participants with self-management tools, including blood pressure cuffs, blood pressure logs, and risk factor goal cards. CMs, physically located in the health care system, introduced patients to self-management skills, prescribed and adjusted medications, and encouraged medication adherence. Meanwhile, in the community, CHWs reinforced self-management skills, served as liaisons between the patient and the health care system, assessed for and assisted in reducing social isolation, and educated patients about stroke risk, especially those related to lifestyle. CMs and CHWs worked collaboratively to address patients’ fluctuating needs, develop and maintain care plans, communicate about patients’ progress, and address barriers as they arose. A physician (vascular neurologist or cardiologist) supervised the CMs and CHWs.

**Figure 1:**
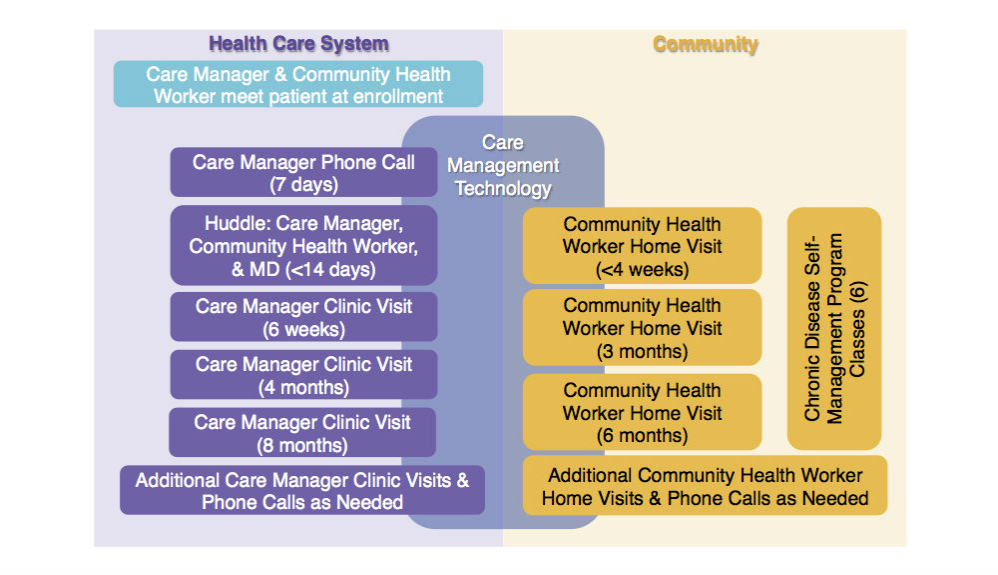
SUCCEED program protocol.

The SUCCEED program protocol (Figure 1) began when a patient was randomized into the program arm and was met by the CM and CHW in the hospital or at an outpatient clinic. The CM followed up with the patient via telephone 1 week after enrollment. During the following week, the CM, CHW, and physician reviewed the patient’s status and jointly developed a care plan. During the course of the year, the patient received clinic visits and home visits by the CM and CHW, respectively. Between visits, patients received phone calls from both the CM and CHW. The patient was also encouraged to attend Chronic Disease Self-Management Program (CDSMP) workshops led by the CHW in community venues [6]. The minimum set of interactions with the patient consisted of three clinic visits and three home visits. Additional interactions took place as needed, particularly for patients with complex care needs.

### SUCCEED Care Team Recruitment and Training

Physicians trained CMs to follow evidence-based protocols and to teach self-management skills and educate patients. CHW training was two-phased. A pool of potential CHWs was recruited through community networks to undertake a 4.5-day training to become certified as CDSMP facilitators. A Master Trainer who had been certified by the Stanford University Program and was affiliated with a local community agency (Watts Labor Community Action Committee) led the training. From the pool of graduates of our CDSMP facilitator training, we recruited individuals to undertake an additional 80 hours of training. The SUCCEED research team collaborated with the Los Angeles Health care Workforce Development Program/Worker Education and Resource Center to develop the curriculum for this 80-hour training on topics specific to SUCCEED, including stroke and vascular risk factors. From the graduates of this longer training, CHWs were hired for the SUCCEED study.

### Design of Care Management Technology for SUCCEED

The CMT was expected to make achieving the target health outcome of decreased stroke risk both more effective and efficient, such that the SUCCEED program could be feasible in other public safety-net settings where resources are highly constrained yet stroke risk factor control is low. SUCCEED researchers worked with a software company to develop the CMT for use by CMs and CHWs, using an existing open-source mobile platform the company developed. The CMT consisted of a CM app containing 22 forms, a CHW app containing 24 forms, and 5 Web-based reports. CMs and CHWs accessed the apps using Android tablets. The CMT was not part of the health system’s electronic medical record (EMR); any information from the EMR that was needed in the CMT had to be manually entered. The CMT was not interfaced with the EMR since the health system was in the process of implementing an EMR during the time that the CMT was being developed.

When the CMT development process began, the SUCCEED program had hired two CMs and two CHWs. All four participated in the development process by developing the content and structure of the forms and reports, testing the forms and reports in the CMT after the software company developed them, and joining regular phone calls with company representatives and SUCCEED researchers. The CMT development process spanned 2 years. The four CMs and CHWs who participated in the development process received in-person training from company representatives. Other CMs and CHWs who were hired later learned how to use the CMT on the job, with assistance from the previously hired and trained CMs/CHWs. All CMs and CHWs received CMT user guides.

**Figure 2:**
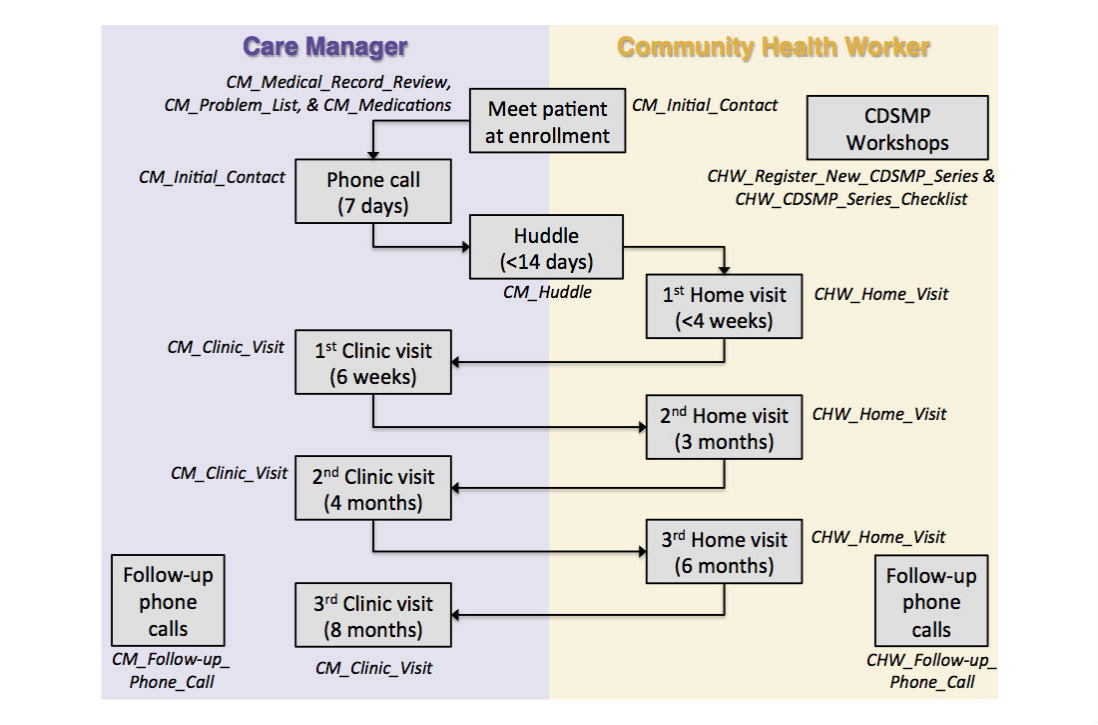
How CMs and CHWs use CMT forms (shown in italics) in the context of the SUCCEED program protocol.

The forms contained in the CM and CHW apps were designed to facilitate patient–CM, patient–CHW, and CM–CHW interactions during each step in the SUCCEED program protocol (Figure 2). The basic structure of the apps and how forms were used in each step is shown in Table 1. For example, CHWs used the CMT during home visits to access forms that guided them in administering depression, self-management, and lifestyle habit assessments. CMs used the CMT to capture patient health information that would then be accessible to CHWs via the CHW app.

In addition to the forms accessed via the apps, CMs and CHWs could access several reports via the platform’s website (Table 2). These Web-based reports were not part of the existing platform but had to be developed specifically for the SUCCEED program. CMs and CHWs could use the reports to view patient-specific care management information, including blood pressure control status, lab results, notes, previous form submissions, completed encounters with patients, and task lists.

## Methods

We conducted qualitative interviews from April to June 2015 with CMs and CHWs in the SUCCEED program.

### Participants

We interviewed all users of the CMT in the SUCCEED program at the time the interviews were conducted, namely two CMs and three CHWs. All were female, one was between 18 and 24 years old, two were between 25 and 34 years old, one was between 35 and 44 years old, one was between 45 and 54 years old, all had at least a high school diploma, and three were Latino. One CM and no CHWs had experience in stroke prevention care management prior to working in the SUCCEED program. Participants had been using the CMT between 9 and 14 months, with an average of 12 months. Both CMs and two of the CHWs participated in CMT development activities. Many of these participant characteristics, most notably, gender, are aligned with the demographic and professional profile of CHWs [7].

### Procedure

We developed the semistructured interview guide based on the Technology Acceptance Model [8] and input from SUCCEED project leaders and the software company’s project manager. The interview consisted of two parts. The first part involved asking CMs and CHWs to demonstrate how they used the CMT to perform predefined key care management activities (Figure 1). Following each demonstration, they were asked to describe their perception of the CMT’s ease of use and usefulness for completing the activity. The second part of the interview probed CM and CHW perceptions of the development process (if applicable), technical support, and the CMT’s information quality, ease of use, usefulness, and impact on CM and CHW roles. Sample interview questions can be found in Multimedia Appendix 1. Interviews were conducted individually (by the first 2 authors), took place in a private room, and lasted an average of 4 hours (range 1-5 hours).

**Table 1:** Structure of CM and CHW applications.

App	Form name	Description
CM	CM_Initial_Contact	Used during the first interaction with a patient after they are enrolled in the program. Used to review contact information, discuss home blood pressure monitoring, and schedule the first CM phone call.
	CM_Medical_Record_Review	Used to capture baseline blood pressure, body mass index, and key lab results. CMs manually populate this form using data from the hospital’s EMR.
	CM_Problem_List	Used to capture a patient’s latest blood pressure, body mass index, and key lab results. CMs manually populate this form using data from the hospital’s EMR. The form is also used to develop a care plan. CMs indicate a patient’s status (at goal, not at goal, not relevant) for each risk factor and can add free-form notes that are visible only to them.
	CM_Medications	A collection of forms, organized by risk factor, that are used to capture medications prescribed to a patient. CMs manually populate these forms using data from the hospital’s EMR.
	CM_First_Phone_Call	Used during the first CM phone call to capture current health status, blood pressure, medication adherence, smoking status, and transportation barriers and to schedule the first clinic visit.
	CM_Huddle	Used during care team huddles to guide discussion about a patient. The form pulls the care plan from the Problem List and allows the team to make revisions.
	CM_Clinic_Visit	Used to guide clinic visits. The form pulls the care plan from the Problem List and allows CMs to make revisions. The end of the form generates a clinic visit summary that CMs can copy and paste into the hospital’s EMR.
	CM_Follow-Up_Phone_Call	Used to document additional CM phone calls. Tasks can also be created in this form.
	CM_Edit_Patient_Schedule	Used to skip the next interaction that is being suggested in the Patient List report.
CHW	CHW_Home_Visit	A collection of forms used to guide home visits. Each form addresses a different topic: stroke literacy, blood pressure, cholesterol, diabetes, antithrombotic use, smoking cessation, depression, diet, physical activity, alcohol and illicit drug use, transportation, communication preferences, and access to care. Forms are organized into 6 sections: assessment, information provision, self-management and adherence, adjustment of medications, clinical support, and resource provision for the patient. Tasks can be created in any of the forms.
	CHW_Review_of_Key_Patient_Data	Used to view the care plan, baseline and latest values for various stroke risk factors, latest medications, which home visit forms have been completed, and a patient’s contact information.
	CHW_Follow-up_Phone_Call	Used to document additional phone calls by the CHW. Tasks can also be created in this form.
	CHW_Register_New_CDSMP_Series	Used to register a new CDSMP workshop series, which consists of 6 weekly sessions. The form captures details such as session dates and meeting location.
	CHW_CDSMP_Series_Checklist	Used during each of the 6 CDSMP sessions to document patient attendance and self-management goals.
CM and CHW	CC_Edit/Update_Patient_Info	Used to update a patient’s home address, preferred language, phone number, family contact information, and primary care physician information.
	CC_Add__Appointment	Used to capture a patient’s appointments with health care providers outside of the SUCCEED care team.
	CC_View/Update_Appointments	Used to view a list of appointments with health care providers outside of the SUCCEED care team, update the appointment details, or close a completed appointment.
	CC_Create_New_Task	Used to create a new task. Tasks can be assigned to another care team member.
	CC_Task_List by_Panel_	Used to view a list of all CM–CHW care team tasks for all patients. The list cannot be filtered by person responsible for completing the tasks (CM or CHW). A task can be revised or closed once it has been completed.
	CC_Task_List_by_Patient	Used to view a list of all CM–CHW care team tasks for a specific patient. The list cannot be filtered by person responsible for completing the tasks (CM or CHW). A task can be revised or closed once it has been completed.

**Table 2:** Description of Web-based reports.

Report name	Description
Patient List	Displays a list of all patients and key data such as date of patient enrollment in program, next suggested interaction per the SUCCEED program protocol, and blood pressure control status.
Patient Information	Displays select data about a patient, including contact information, primary care physician information, lab results, and notes.
Form Submissions	Displays a list of all forms that have been submitted for a specific patient. Forms on the list can be opened to view a readable version.
Interactions with Patients	Displays, at the patient level, the minimum set of interactions with patients and their target completion dates per the SUCCEED program protocol. When an interaction has been completed, the report shows the date of completion and the name of the care team member who completed it. The report displays an alert when an interaction is overdue. Additionally, it provides a snapshot of risk factor control status.
Care Management Tasks	Displays a list of all CM–CHW care team tasks for all patients. The list can be filtered by person responsible for completing the tasks (CM or CHW) and by patient. A task can be revised or closed once it has been completed.

### Data Analysis

Interviews were audio recorded and transcribed verbatim, resulting in 500 pages of transcripts. Dedoose version 7.1.3 (SocioCultural Research Consultants) was used to manage and code the data. Two members of the research team (the first 2 authors) developed an initial coding scheme that included a priori codes derived largely from literature on the impact of HIT on workflow, unintended consequences of HIT, technology acceptance, and care coordination models [9-13]. Three coders (including the first author) then coded 60 pages of transcripts independently. Coding discrepancies were discussed with the second author until consensus was reached. New codes were added and code definitions were adjusted for clarity. The same individuals independently coded the remaining pages of transcripts (each page was coded by 2 individuals) and met regularly with the second author to discuss and resolve any coding discrepancies. The coded excerpts were grouped into broader themes and then related in a conceptual model of how the CMT facilitated care management activities. These interpretations were shared with the larger research team for final review.

### Patient Survey

We conducted a patient survey in order to obtain the perspective of patients regarding CHWs’ use of tablets during home visits. After patients completed the SUCCEED exit interview, they were given an informational flyer that invited them to participate in a new research study. Interested patients consented to being called by a research assistant who explained that the research study entailed a one-time survey. Patients were eligible to participate in the survey if they received at least two home visits from a SUCCEED CHW. If patients were eligible, they met in person with the research assistant who administered the survey in English or Spanish. All patients provided written informed consent. The survey consisted of 16 Likert-type questions. Examples are, “To what extent do you agree or disagree that the tablet computer helped the CHW explain your condition and how to care for it?” and “To what extent do you agree or disagree that the CHW’s use of a tablet computer made home visits feel less personal?” At the end of the survey, patients were asked if they wanted to comment on the CHW’s use of a tablet during home visits. Responses were recorded verbatim. The Institutional Review Boards at the University of California, Los Angeles, University of Southern California, and Rancho Los Amigos National Rehabilitation Center approved the patient survey procedures. Patients who completed the survey received a US $20 gift card.

## Results

CMs and CHWs described their perceptions on the usefulness and challenges of the CMT’s intended functions (summarized in Table 3). In addition, they discussed broader usability issues of the beta-version CMT that disrupted their use of the tool. Patient responses to the survey of CHW tablet use are described.

### Care Management Technology Usefulness and Challenges

**Figure 3:**
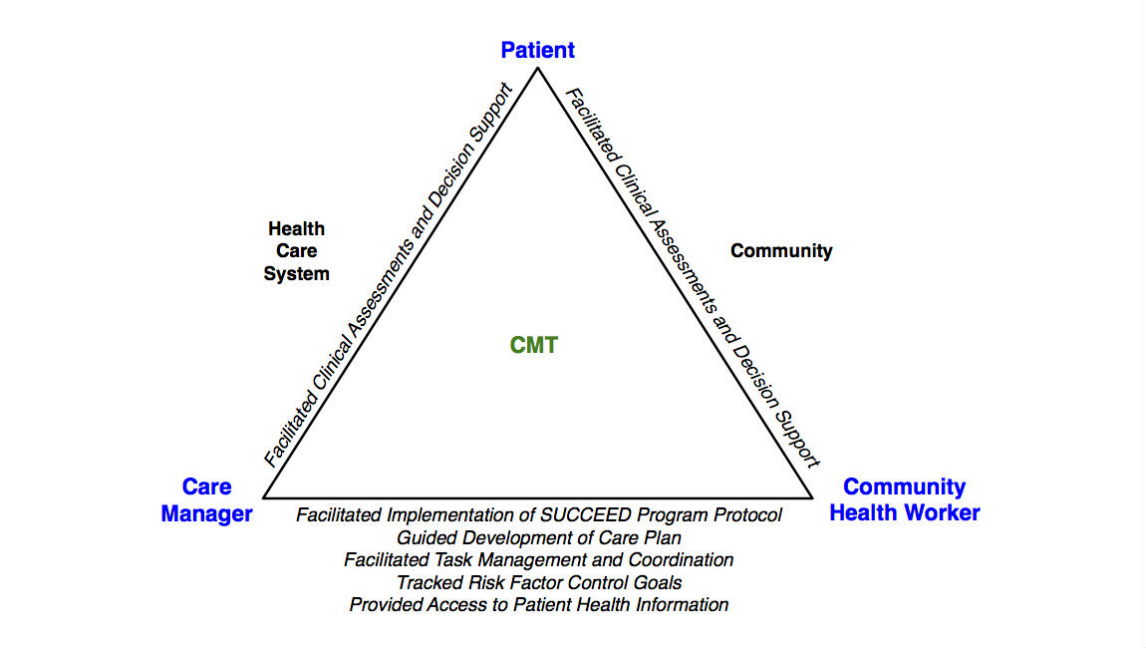
A conceptual model showing CM and CHW perceptions of how the CMT facilitated care management activities involving interactions between three key players: patients and CMs, patients and CHWs, and CMs and CHWs.

Overall, CMs and CHWs expressed that the CMT was useful in helping them to perform key aspects of stroke prevention care management (Figure 3). Statements by CMs and CHWs indicated that the CMT helped them keep track of their interactions with patients and plan their work accordingly to ensure that patients received the minimum set of interactions per the SUCCEED program protocol. CMs thought the CMT was helpful for developing care plans and sharing these plans with CHWs in the field. CHWs reported that the CMT enabled electronic data collection of clinical assessments and provided decision support when performing patient education and self-management training. According to CHWs, the CMT was helpful for tracking patients’ stroke risk factor values and accessing these values and other important patient-specific care management information remotely during home visits. Finally, CMs and CHWs explained how the CMT had necessary features in place for facilitating task management and coordination—namely, documenting work by creating personal tasks and tasks for other team members, tracking tasks, viewing task lists—but cited usability problems as a barrier to use.

CMs and CHWs experienced several challenges when using the CMT. The number of clicks and screen changes needed to create, update, or close tasks deterred CMs and CHWs from using the CMT for task management. Additional issues resulted from limitations of the platform, including the inability to link to educational materials outside the platform or to generate graphics of risk factor values over time. Finally, issues with the design of the CMT’s content—extensive use of free-text responses, lengthy forms, and especially the manual transfer of data from the EMR to the CMT—severely hindered CM and CHW efficiency.

### Care Management Technology Usability Issues

The main challenge to using the CMT was long loading times, as evidenced by the numerous statements from both the CMs and CHWs expressing their frustration. The slow response times created an unpleasant user experience. As one CHW stated, “[The CMT] is so painfully slow.” It hindered CM and CHW productivity because they could not move freely and keep their attention focused on the task at hand; they reported performing other tasks while waiting for the CMT to finish loading. The consequence was that some participants bypassed the CMT entirely for certain care management activities, developing their own ways of accomplishing the work with greater efficiency. CMT reliability was another barrier that was reported. It was not uncommon for the CMT to be down for an unspecified period of time and without warning. CM workflow was significantly affected, especially if it happened during clinical huddles or clinic visits. One CM said that the CMT’s reliability issues made her feel “anxious and vulnerable” during clinical encounters. For this reason, she reported printing CMT form submissions as a backup.

**Table 3:** CM and CHW perceptions on the usefulness and challenges of the CMT’s intended functions.

Intended CMT functionality	Usefulness	Challenges
Facilitate implementation of the SUCCEED program protocol	Provided list of patient interactions, suggested completion dates, and provided actual completion dates	Decreased efficiency by increasing amount of steps needed to extract certain types of information
	Allowed CMs to view completed and pending CHW interactions (and vice versa)	Did not always have accurate or complete information of patient interactions
	Provided dates that helped with prioritizing patients with upcoming graduations in order to meet minimum required patient interactions	Did not allow CMs and CHWs to add additional patient interactions beyond minimum set nor change order in which they wanted interactions to occur/be displayed
Guide development of care plans	Enabled CMs to indicate whether each risk factor was controlled	Did not specify who could add and view free response notes, causing confusion among CMs and CHWs about how to use free response notes
	Enabled CMs to display care plans during huddles and make any necessary adjustments in real time	
	Reminded CMs and CHWs what risk factors to focus on during clinic and home visits, respectively	
Facilitate task management and coordination	Enabled CMs and CHWs to create personal tasks and tasks for other team members, track tasks, and view task lists	Required undue effort to create task
		Did not allow CMs and CHWs to filter tasks by person responsible when viewing task list using app
Facilitate clinical assessments and provide decision support	Eliminated paper forms and manual documentation during home visits	Contained lengthy forms that made form navigation difficult
	Guided CHW-patient conversations and tailored forms in real time	Created additional steps in workflow because it did not directly link to educational materials
	Prompted CHWs to take specific actions to address patients’ unique needs	Made extensive use of fields requiring free-text responses
Track risk factor control goals	Capable of tracking patients’ risk factor values	Did not display or provide easy access to all of patients’ risk factor values collected over time
	Displayed patients’ baseline and most recent risk factor values	Could not generate useful visualizations to display for patients
Provide access to patient health information	Enabled CHWs to immediately access patient health information during home visits	Required CMs to manually transfer data between CMT and EMR, which was time consuming, subject to errors, and difficult to keep current
		Contained incomplete information for CHWs during home visits when CMs had not completed data entry

### Patient Perspective of Community Health Workers’ Tablet Use

Out of 25 who were approached and consented to being contacted by the research assistant, 14 patients completed the survey. Patient characteristics and responses to each item in the survey are shown in Multimedia Appendix 1. The average age of survey respondents was 54 years, 71% (10/14) were male, and 64% (9/14) were Latino. Overall, patients viewed favorably CHWs’ use of a tablet during home visits. For example, all patients agreed or strongly agreed that the tablet helped the CHW explain their condition and how to care for it. All patients agreed or strongly agreed that they were comfortable talking to the CHW about their health when she used the tablet, and only one patient agreed that the CHW’s use of a tablet made home visits feel less personal. Furthermore, several patients described specific ways in which the tablet computer enhanced the CHW-patient interaction during home visits: ability to view patients’ prescribed medications and lab values, guide which questions to ask patients, reduce paper-based documentation, and print patient handouts.

## Discussion

### Principal Findings

The overall assessment by CMs and CHWs was that the CMT was useful in terms of its intended functions of facilitating implementation of the SUCCEED program protocol, guiding development of care plans, facilitating clinical assessments and providing decision support, tracking risk factor control goals, and providing access to patient health information. On the other hand, CMs and CHWs encountered usability problems that made it difficult to use the tool as it was intended for task management and coordination. In addition, problems with the CMT’s performance had a substantially negative effect on the user experience.

The CMT functionalities that CMs and CHWs in this study found to be useful are aligned with some of the ways it was proposed in a systematic review that HIT could support effective collaborative care [3]. These included providing data about patient interactions and highlighting interactions that have not occurred, making the care plan visible and allowing it to be shared across care team members, enabling care team members to document clinical data, making clinical data accessible to all members of the care team (not just clinicians), and providing decision support. Providing alerts for patients who are not improving has also been suggested as an HIT capability for supporting collaborative care models, but this was not mentioned as a potentially useful CMT functionality by CMs and CHWs in our study. Given the promise of using electronic provider alerts to effectively improve blood pressure control [14], the next iteration of the CMT design could include alerts for when patients’ risk factor values are not at goal and future research could examine CM/CHW acceptance and impact of these alerts.

Like CMs and CHWs in our study, there is consensus among experts that important HIT components for supporting team-based models of chronic illness care include the ability to conduct clinical assessments and surveys, display a patient’s progress in terms of the long-term treatment plan, and provide graphs of outcomes over time to show patients [15]. Unlike our findings, experts have also identified as important the ability to present a history of treatment. Perhaps the CMs in our study did not mention this as a desirable CMT feature because the information was available in the EMR, which was used alongside the CMT when needed. Nonetheless, it may be helpful if health care and community partners alike can easily determine what everyone in the care team is currently doing and has previously done [16], which is information that may not necessarily be available in the EMR.

### Lessons Learned

We summarize the key lessons that we learned from designing, developing, and implementing a CMT in the SUCCEED program. Our experience may help other researchers who are also considering customizing an existing platform for use in studies of how CMTs enhance team-based care for chronic disease management.

#### Lessons 1: Ask Vendor Key Questions

Prior experience indicates that it is best to ask potential vendors to walk through how their product would be used to accomplish predefined scenarios [17]. However, when scenarios cannot be demonstrated because no existing platforms have the desired functionality, as was the case for the SUCCEED program, we determined critical questions to ask during the vetting process. We asked potential vendors whether they could develop additional features to meet the needs of the program, and we incorporated that scope of work into the contract. If we could do this process over, we would negotiate and agree on what would happen if the development of these new features negatively affected the system. Are the vendor’s efforts to fix these potential problems included in the cost of the system? How soon after problems are discovered will the vendor look into them to figure out a solution? The contract would then specify, as the National Learning Consortium has recommended [17], the conditions under which a breach of contract has occurred.

#### Lessons 2: Carefully Select Essential Data That Should Go Into Care Management Technology

We also learned that it is vital for the data entered into the CMT to complement rather than duplicate data that are already available in the health system’s EMR. A systematic review found that mobile technologies are most commonly used by CHWs to collect health data [18]. However, SUCCEED CHWs needed to be able to both collect and retrieve data while in the field. There was no guidance in the literature about the necessary data elements to include in the CMT for CHW retrieval. We had intended for CMs to use the CMT to document and share with CHWs only key pieces of data needed for CHW work. However, we lost sight of this vision in the midst of the long process of customizing the platform. Consequently, some CM data collection forms ended up requiring that CMs enter information from the EMR that was not essential. This adversely affected their efficiency given that they had to manually transfer the data. Until CMTs and EMRs are compatible, which we predict will eventually happen, careful selection of which essential data should go into the CMT will help to reduce duplication.

#### Lessons 3: Engage More Experienced End Users in the Design Process

We learned that it is important to engage more experienced end users in the design process to think critically about the necessary CMT components, and for project leaders to make a distinction between must-have versus nice-to-have components. Indeed, the user-centered design literature recommends involving users with high levels of competence and experience [19]. However, because the SUCCEED program was a new model of care, the end users who were actively involved in designing the workflows and the CMT did not have experience with the type of care management and care coordination that would be required. Instead, these CMs and CHWs, working with the project’s scientific and clinical leaders, designed both the workflows and the CMT based on a projection of what would be needed. The lack of experience of the end users—a consequence of this being a new approach to care—partially contributed to building a system that was more complicated than necessary. Because platforms may have a limited amount of processing power, design teams should err on the side of caution and focus on developing the must-haves first. Then, if the CMT is performing as expected, teams can work on the nice-to-haves, as time and resources permit.

#### Lessons 4: Apply a Structured, Iterative Approach When Improving the Care Management Technology

After implementation, CMs and CHWs reported to the vendor performance issues they encountered when using the CMT. Generally, the vendor would plan a fix and often but not always communicate with the end users when the fix had been implemented. In addition, there was not a closed loop, systematic tracking mechanism to determine the effects of the fix for end users, including both intended and unintended effects. We need to apply a structured, iterative approach when improving the CMT to assess whether the changes actually produced the desired results. The plan-do-check-act cycle could serve as a model [20]. When an issue is reported and the vendor plans and communicates the timing of implementing a fix, the vendor, end users, and project leaders would all agree on tracking data and a process for assessing the impact of the fix. The vendor would plan further improvements if the feedback indicated that they were necessary. Ideally, such performance issues would be addressed during the development phase instead of after the CMT has been implemented.

### Limitations

This study was limited to a small sample of CMT users, some of whom also helped design the SUCCEED CMT. Thus, the interviewed participants may not necessarily represent the larger user population. However, the sample size is consistent with findings from user-experience research indicating that 5 users can detect most problems [21]. Additionally, we believe that participants’ experience with using the CMT prior to this study enabled them to readily assess the CMT’s usefulness. A second limitation was that the CMT evaluated in this study was designed specifically for stroke prevention. The findings about useful CMT components may therefore not be generalizable to CMTs for other chronic conditions. Nonetheless, care management and care coordination for people with multiple health and social needs, such as the patients in the SUCCEED program, share similar elements [8], suggesting that our findings of perceived useful components may also be useful to care teams beyond stroke prevention. A third limitation is that the CMT evaluation involved a product in its beta stage of development. There were no guidelines regarding the range of CMT functionalities needed for collaborative, team-based models of care. The development team erred on the comprehensive side and hence may have caused more usability issues than a mature product would present.

### Conclusions

The CMT used in the SUCCEED program is an initial step toward the development of effective HIT tools to support collaborative, team-based models of care. The CMs and CHWs we interviewed generally perceived the CMT as useful for stroke prevention care management. Our findings suggest useful functionalities of CMTs supporting health care and community partners. These functionalities include enabling users to collect clinical assessment data from patients, access patient information, develop a care plan, track implementation of the program protocol, track risk factor control goals, manage and coordinate care tasks, and receive decision support during patient interactions. Efforts to implement CMTs, however, will be futile unless usability issues are properly addressed during the development process. The CMT will need to be modified as the evidence base grows. Future research should go beyond user perceptions of the CMT’s usefulness by assessing the CMT’s effect on team performance. If the SUCCEED intervention is shown, once the trial is completed, to improve stroke risk factor control, we anticipate disseminating this model of care broadly and encouraging uptake in safety-net health care settings that are implementing team-based models of chronic illness care.

## Appendix

Multimedia Appendix 1 CM and CHW interview questions, patient characteristics, and patient survey results.

## Abbreviations

CDSMP: Chronic Disease Self-Management Program
CHW: community health worker
CM: care manager
CMT: care management technology
EMR: electronic medical record
HIT: health information technology
SUCCEED: Secondary Stroke Prevention by Uniting Community and Chronic Care Model Teams Early to End Disparities
