# Supplementary material for: Using Beta-Version mHealth Technology for Team-Based Care Management to Support Stroke Prevention: An Assessment of Utility and Challenges
Source: JMIR Res Protoc. 2017 May 23;6(5):e94. doi: 10.2196/resprot.7106 (PMC5461415; doi:10.2196/resprot.7106)
Supplement: Multimedia Appendix 1 [file resprot_v6i5e94_app1.pdf]

Appendix

Observation Questions

After observing the user perform a key care management activity with the CMT, ask the following questions:

Perceived Ease of Use

1. How easy or difficult is it to use the CMT to complete this activity?

2. How do you feel about the amount of time it takes to complete this activity using the CMT?

3. What are your thoughts on the completeness and accuracy of information on the CMT for this activity?

Perceived Usefulness

4. What are some of the advantages and disadvantages of using the CMT over a paper-based system for completing this task?

Challenges and Opportunities

5. What do you find challenging about using the CMT to complete this activity?

6. If you could make a significant change to the CMT for completing this activity, what would it be and why?

Interview Questions

The following questions are in regards to your overall experience using the CHW (or CM) app and the SUCCEED custom reports.

Development Process (only if applicable)

Having gone through the process of helping to develop the forms and the structure of the CMT…

1. To what extent does the CMT operate how you thought it would?

2. Is there anything about the CMT that turned out not to be as useful in practice as you originally thought it would during the development process? If so, what?

3. What are your thoughts on the overall process of developing the forms and structure of the CMT?

    a. Probe: What are your thoughts on the ease of access to the vendor representative that worked with the team to develop the CMT for the SUCCEED trial?

Usability

4. Overall, how easy or difficult is it to use the CMT?

5. How easy or difficult was it to learn how to use the CMT?

6. Can you describe your experience with the training you received to learn how to use the CMT?

    a. Probe: What did you find most helpful?

    b. Probe: How do you think the training can be improved?

7. How comfortable do you feel using the CMT?

8. To what extent do you feel like you understand the purpose of all the various components in the CMT?

9. Overall, how does the CMT affect the time it takes you to do your work?

10. When you make a mistake on the CMT, how easy or difficult is it to correct the mistake or to recover and continue with the correct workflow on the device? Please describe any examples.

Information Quality

11. What are your thoughts on the completeness and accuracy of information on the CMT for helping patients to control their stroke risk factors?

12. How easy or difficult is it to understand the information provided with the CMT?

13. What are your thoughts on how the information in the CMT is organized?

14. How familiar are you with the structure of each report and form in the CMT?

15. How easy or difficult is it to find the information you need in the CMT?

16. To what extent is the information you capture on the CMT <u>necessary</u> for the care management of your patients?

    a. Probe: What specific information do you feel is not necessary? Why not?

17. To what extent is the information you capture on the CMT useful for the care management of your patients?

    a. Probe: What specific information is not useful?

Perceived Usefulness and Impact on CHW (or CM) Roles

18. Overall, how has using the CMT made a difference in or had an impact on or affected carrying out your role as a CHW (or CM)?

    a. Probe: How has using the CMT affected self-management and behavior change education and training?

    b. Probe: How has using the CMT affected your face-to-face communication with patients?

    c. Probe: How has using the CMT affected your speed and effectiveness in communicating with CMs (or CHWs) about shared patients?

19. What components of the CMT do you find most and least useful? Why?

20. To what extent is the CMT useful for determining your work priorities for the day or the week? Examples of work priorities could be scheduling visits, completing the visits, working on tasks, etc.).

    a. Follow up: How easy or difficult is it to determine your work priorities?

    b. Follow up: How easy or difficult is it to find a specific patient or task?

Utilization

21. Will you explain how you make decisions about which forms to complete for each patient?

Attitude Toward Using

22. Overall, how do you feel about using the CMT versus using a paper-based system?

23. Do you trust that the data you enter in the CMT is saved and reliably accessible?

Intention to Use

24. If you were given the choice between using the CMT or a paper-based system, which one would you use? Why?

Technical Support

25. What do you do if encounter technical problems while using the CMT?

    a. Probe: Whom do you contact?

    b. Probe: What resources are available for resolving these problems?

Wrap-up

26. Would you recommend other CHWs to use the CMT or a paper-based system? Why?

27. Is there anything else you would like to tell us about your experience using the CMT?

Table A-1 Patient Characteristics

Characteristics	Total (N=14)
Age, M (SD)	Mean=54 years (Std=8.5 years)
Male	71% (10/14)
Race	
American Indian / Alaskan Native	7% (1/14)
Asian	14% (2/14)
African American	14% (2/14)
Caucasian	64% (9/14)
Ethnicity: Hispanic	64% (9/14)
Born in the United States	21% (3/14)
Living with at least one other adult	92% (13/14)
Education	
Some college	14% (2/14)
At least high school graduate or equivalent	21% (3/14)
Some high school	28% (4/14)
8th grade or less	35% (5/14)
Working for pay, part- or full-time, prior to stroke	35% (5/14)

Table A-2 Results from patient survey (N=14)

Statements	Not at all	A little	Some	A lot	Don't know
How much did the community health worker use the tablet computer to ask you questions about your condition?	0 (0.0%)	0 (0.0%)	3 (21.4%)	11 (78.6%)	0 (0.0%)
How much did the community health worker use the tablet computer to show you educational information or videos?	1 (7.1%)	2 (14.3%)	2 (14.3%)	8 (57.1%)	1 (7.1%)
How much did the community health worker use the tablet computer to look up information to address your concerns?	0 (0.0%)	0 (0.0%)	4 (28.6%)	10 (71.4%)	0 (0.0%)
	Strongly disagree	Disagree	Neither disagree or agree	Agree	Strongly agree
Overall, I am satisfied with the explanations I received from the community heath worker about my condition and how to care for it.	0 (0.0%)	0 (0.0%)	0 (0.0%)	5 (35.7%)	9 (64.3%)
The tablet computer helped the community health worker explain my condition and how to care for it.	0 (0.0%)	0 (0.0%)	0 (0.0%)	4 (28.6%)	10 (71.4%)
The tablet computer made the community health worker take longer to explain my condition and how to care for it.	5 (35.7%)	6 (42.9%)	0 (0.0%)	1 (7.1%)	2 (14.3%)
I am satisfied with the communication I had with the community health worker during the home visit.	0 (0.0%)	0 (0.0%)	0 (0.0%)	3 (21.4%)	11 (78.6%)

Because of the tablet computer, the community heath worker spent less time listening to me than I would have liked during the home visit.	6 (42.9%)	6 (42.9%)	1 (7.1%)	1 (7.1%)	0 (0.0%)
I was comfortable talking to the community health worker about my health when she used a tablet computer.	0 (0.0%)	0 (0.0%)	0 (0.0%)	3 (21.4%)	11 (78.6%)
Because of the tablet computer, the community health worker spent less time talking with me than I would have liked during the home visit.	2 (14.3%)	8 (57.1%)	2 (14.3%)	2 (14.3%)	0 (0.0%)
The community health worker's use of a tablet computer made home visits feel less personal.	2 (14.3%)	9 (64.3%)	2 (14.3%)	1 (7.1%)	0 (0.0%)
Because of the tablet computer, the community health worker spent less time looking at me than I would have liked during the home visit.	2 (14.3%)	8 (57.1%)	4 (28.6%)	0 (0.0%)	0 (0.0%)
I believe my personal information was safe in the tablet computer used by the community health worker.	0 (0.0%)	1 (7.1%)	0 (0.0%)	3 (21.4%)	10 (71.4%)
The community health worker's use of a tablet computer made the home visits take longer than I would have liked.	2 (14.3%)	8 (57.1%)	2 (14.3%)	1 (7.1%)	1 (7.1%)
I believe the tablet computer helped to ensure that the community health worker provided the care I needed.	0 (0.0%)	1 (7.1%)	0 (0.0%)	5 (35.7%)	8 (57.1%)
I did not like the idea of the community health worker using a tablet computer during home visits.	4 (28.6%)	8 (57.1%)	2 (14.3%)	0 (0.0%)	0 (0.0%)
